# Focus On: Women and the Costs of Alcohol Use

**DOI:** 10.35946/arcr.v35.2.12

**Published:** 2014

**Authors:** Sharon C. Wilsnack, Richard W. Wilsnack, Lori Wolfgang Kantor

**Affiliations:** **Sharon C. Wilsnack, Ph.D.,***and***Richard W. Wilsnack, Ph.D.,***are both professors in the Department of Clinical Neuroscience, University of North Dakota School of Medicine & Health Sciences, Grand Forks, North Dakota.*; **Lori Wolfgang Kantor, M.A.,***is a science editor at alcohol Research: Current Reviews.*

**Keywords:** Alcohol consumption, alcohol use, abuse, and dependence, alcohol use disorder, alcohol burden, drinking patterns, prevalence, alcohol burden, alcohol-related problems, alcohol-related injuries, women, pregnancy, cardiovascular disease, stroke, bone mass density, breast cancer, liver disease, psychiatric disorders, posttraumatic stress disorder (PTSD), depression, eating disorders, suicidal behavior, intimate partner violence, sexual assault

## Abstract

Although light-to-moderate drinking among women is associated with reduced risks of some cardiovascular problems, strokes, and weakening of bones, such levels of drinking also are associated with increased risks of breast cancer and liver problems, and heavy drinking increases risks of hypertension and bone fractures and injuries. Women’s heavy-drinking patterns and alcohol use disorders are associated with increased likelihood of many psychiatric problems, including depression, posttraumatic stress disorder, eating disorders, and suicidality, as well as increased risks of intimate partner violence and sexual assault, although causality in the associations of drinking with psychiatric disorders and with violence remains unclear. It is important for women to be aware of the risks associated with alcohol use, especially because gaps between U.S. men’s and women’s drinking may have narrowed. However, analyses of health risks and benefits need improvement to avoid giving women oversimplified advice about drinking.

Even though the prevalence of alcohol use in the United States generally is lower among women compared with men (Substance Abuse and Mental Health Services Administration [SAMHSA] 2011), this gap has narrowed (Grucza et al. 2008). Furthermore, although women consume alcohol at lower levels than men, their body composition puts them at higher risk than men of developing some alcohol-related problems, both acutely (because of higher blood alcohol levels from a given amount of alcohol^1^) and chronically (from alcohol-related organ damage). This article examines alcohol-use patterns (with particular attention to midlife) and how they differ for men and women and summarizes recent evidence on associations between women’s alcohol consumption and their physical and mental health.

## Drinking Practices and Patterns Among Women in Midlife

Rates of drinking decline with age for both men and women in the United States, and drinking remains less prevalent among women compared with men. In 2010, the proportion of people reporting at least one drink in the previous 30 days (i.e., current drinkers) decreased from 70 percent among 21- to 25-year-olds to 61.1 percent among 40- to 44-year-olds and 51.6 percent among 60- to 64-year-olds (SAMHSA 2011). The same survey also found that approximately 57.4 percent of males aged 12 or older were current drinkers, compared with 46.5 percent of females of the same age range (SAMHSA 2011).

Rates of binge drinking also are higher among men than women (Centers for Disease Control and Prevention [CDC] 2012). One survey (National Institute on Alcohol Abuse and Alcoholism [NIAAA] 2012) reported that 28.8 percent of women and 43.1 percent of men reported binge drinking (i.e., consuming within 2 hours four or more drinks for women and five or more drinks for men) in the previous year. In a multinational study of 35 countries, Wilsnack and colleagues (2009) reported that, as expected, men consistently drank more than women and were more likely to engage in high-volume drinking and high-frequency drinking. Women were more likely to be lifetime nondrinkers and to be former drinkers.^2^ The authors suggest that women may find it easier than men to quit drinking because (1) women generally are lighter drinkers than men; (2) drinking is not as important to women’s social roles as it is to men’s; and/or (3) women who stop drinking during pregnancy and early childrearing may not resume drinking later on.

Despite these findings, Grucza and colleagues (2008) reported significant increases between 1990–1991 and 2000–2001 in the lifetime prevalence of drinking for women aged 38–47 in the United States. There also was an increase in lifetime prevalence of alcohol dependence among women drinkers aged 38-47. Similar increases were not found for male drinkers, suggesting that the gender gap in alcohol use and dependence is narrowing, at least in these age groups.

## Drinking During Pregnancy: Patterns and Predictors

Women who become pregnant in their thirties and forties may be more likely to drink during pregnancy than younger women. From 2001 to 2005, 17.7 percent of 35- to 44-year-old women reported drinking during pregnancy, compared with 8.6 percent of pregnant women aged 18–24 (Denny et al. 2009). Among women in eight States who gave birth between 1997 and 2002, 30.3 percent reported drinking during pregnancy, and 8.3 percent reported binge drinking (four or more drinks on one occasion). Whereas 22.5 percent of the women reported drinking during the first month of pregnancy, drinking declined during pregnancy; only 7.9 percent of women reported drinking during the third trimester, and only 2.7 percent reported drinking during all trimesters. Drinking during pregnancy was more prevalent among women over 30 (more than 30 percent drank) than among younger women (Ethen at al. 2009).

Understanding the predictors of drinking during pregnancy may help target prevention efforts. The eight-State study by Ethen and colleagues (2009) found that both drinking and binge drinking during pregnancy were predicted by prepregnancy binge drinking. Drinking and binge drinking during pregnancy also were more prevalent among women who were non-Hispanic whites, who smoked during pregnancy, and whose pregnancy was unintended. A recent review of 14 studies of drinking during pregnancy in nine countries (Skagerstróm et al. 2011) found that drinking during pregnancy was associated with heavier drinking prior to pregnancy in all seven studies that measured this; smaller numbers of studies consistently found that drinking during pregnancy was associated with higher income/social class and with histories of abuse or exposure to violence and histories of drinking problems.

## Physical Health Effects of Women’s Drinking

Light to moderate alcohol use has been found to generally be beneficial for many health outcomes and is associated with decreased mortality. Heavier use, however, is associated with poorer health and increased mortality. One meta-analysis of 34 studies in 13 countries found that, compared with abstaining, drinking less than two drinks per day among women and drinking less than four drinks per day among men was associated with significantly reduced total mortality, but higher levels of alcohol use were associated with increased mortality (Di Castelnuovo et al. 2006). These findings should not encourage people to start drinking alcohol for its health benefits, because of the significant health problems associated with heavier use, as described below.

Another study used data from a large survey of middle-aged (median age 58) female nurses in the United States and assessed the health of participants who lived to age 70 and older. The study found that light to moderate alcohol consumption at midlife was associated with modestly increased odds of good health at age 70 or older (no chronic illnesses, physical impairment, or mental problems). That is, women who averaged between one-third and one drink per day had about 20 percent higher odds than nondrinkers of good health at age 70 and older. Also, the women who drank frequently during the week (5 to 7 days) had better odds of good health at age 70 and older than the women who drank only once or twice a week (Sun et al. 2011). However, these findings should be interpreted with caution because the measures of alcohol consumption were quite limited.

### Effects of Women’s Drinking on Cardiovascular Health

Many studies have found that light to moderate alcohol use is associated with lower risks of cardiovascular disease and mortality, but these studies often have not reported specifically on women’s drinking. However, studies of coronary heart disease risk in Denmark (Tolstrup et al. 2006) and England (Ward et al. 2011) found that the risks were lower in women who consumed more alcohol. In the United States, pooled data from nine National Health Interview Surveys (1987–2000) showed that women drinking up to seven drinks per week had lower risks of cardiovascular mortality than lifetime abstainers (Mukamal et al. 2010).

Light-to-moderate drinking also may be associated with lower risks of sudden cardiac death (SCD). The study of nurses in the United States, which examined heart problems in 4-year periods after reported drinking or abstaining, found the lowest risk of SCD among women who averaged approximately one-half to one drink per day. Women who drank more heavily (more than 30 g or two drinks per day) had SCD risks similar to risks of abstainers, but the number of SCD cases among women who consumed more than 30 g per day was limited (Chiuve et al. 2010). As noted earlier, however, these findings are based on limited measures of drinking.

In contrast to studies finding beneficial effects, a meta-analysis of six studies (Samokhvalov et al. 2010) found that women’s risks of atrial fibrillation (AF) increased steadily with increasing alcohol consumption. Whereas women who averaged up to two drinks a day did not have significantly higher risks than abstainers, women who consumed more than two to three drinks daily had a 17 percent increased risk of AF, and women who consumed more than four drinks daily had twice the risk of AF.

Women’s risk of hypertension also may increase steadily as alcohol consumption increases. A meta-analysis of eight studies indicated that the risk was reduced somewhat among women drinking lightly (averaging less than a drink a day), but the risk then rose steadily with higher levels of consumption. Compared with abstainers, women who averaged roughly four drinks a day had nearly twice the risk of hypertension, and women averaging roughly eight drinks a day had nearly three times the risk (Taylor et al. 2009).

### Effects of Women’s Drinking on Stroke Risk

The risk of stroke is lower among women who are light-to-moderate drinkers. The U.S. nurses’ study found lower risk of strokes among women who were recent light drinkers, averaging approximately one drink a day (Jimenez et al. 2012). Among 45,449 Swedish women aged 30 to 50 who were followed up approximately 11 years later, risks of ischemic stroke were significantly lower among women averaging less than one drink a day (compared with abstainers). The numbers of women with hemorrhagic strokes and/or strokes after drinking more heavily were too small for reliable evaluation (Lu et al. 2008). Meta-analyses of five to nine other studies found that women’s light-to-moderate drinking was protective against both ischemic and hemorrhagic strokes (with lowest risks in women averaging about one drink a day), but risks of morbidity and mortality from both types of strokes increased rapidly as women’s consumption rose above three to four drinks a day (Patra et al. 2010).

### Effects of Women’s Drinking on Liver Disease

Women apparently are more vulnerable than men to liver cirrhosis and other liver injury from alcohol use, possibly because of estrogens, although the mechanisms are as yet unclear (Eagon 2010). A meta-analysis of 12 studies found that women’s risks of morbidity and mortality from liver cirrhosis increased steadily with higher levels of alcohol consumption, with no protective effect of light to moderate drinking, and the risks increased more rapidly for women than for men (Rehm et al. 2010). These risks may be increased by other personal characteristics and by drinking patterns. In a very large sample of women in the United Kingdom, followed up for an average of 6.2 years, risks of cirrhosis among women averaging two or more drinks a day increased greatly if their body mass indexes were greater than 28 kg/m^2^ (Liu et al. 2010). In a large study of women in New York State, levels of γ-glutamyl-transferase (GGT), a liver enzyme that increases in all forms of liver disease (Niemelä and Alatalo 2010), were highest not only in women who averaged more than a drink a day but also in women who did their drinking only on weekends and without food (Stranges et al. 2004).

### Effects of Women’s Drinking on Breast Cancer Risk

Even moderate alcohol consumption increases breast cancer risk, and the risk rises as drinking increases. A multinational meta-analysis of 98 studies found that the risk of breast cancer increased an average of 10 percent for every increase of 10 grams per day in alcohol consumption (Key et al. 2006). A 10-year follow-up study of more than 38,000 U.S. women aged 45 and older found a significant trend of increased risk of invasive breast cancer associated with increased alcohol consumption at baseline, with the greatest risk among women averaging at least 30 grams of alcohol per day (Zhang et al. 2007). The risks from alcohol consumption were clearest for estrogen- and progesterone-receptor-positive tumors and for women currently taking postmenopausal hormones, consistent with the hypothesis that part of alcohol’s effect on breast cancer is to increase estrogen exposure (Garcia-Closas et al. 2002; Onland-Moret et al. 2005). Another U.S. study, based on data from 184,418 postmenopausal women aged 50 to 71, reported similar findings (Lew et al. 2009). After 7 years of follow-up, the researchers found that risks of breast cancer increased steadily the more women drank. Risks were highest for estrogen- and progesterone-receptor–positive tumors, with risks of these tumors 46 percent higher for women drinking more than 35 grams of alcohol (more than two drinks) a day. However, when Suzuki and colleagues (2010) followed up 50,757 Japanese women (aged 40 to 69) over 13 years, they found that breast cancer risk increased 6 percent with every additional 10 grams per day of alcohol consumption, but the observed association was not modified by menopausal status or use of exogenous estrogens. These findings suggest that breast cancer risks associated with alcohol consumption involve more than just estrogen levels.

### Effects of Women’s Drinking on Bone Health

Higher bone-mineral density (BMD) is associated with resistance to fracture. A recent review of research relevant to 40- to 60-year-old women concluded that there was fair evidence that moderate drinking did no harm to BMD (Waugh et al. 2009). In fact, a number of studies have found that light to moderate drinking is associated with increased BMD, at least among postmenopausal women (Maurel et al. 2012). For example, Tucker and colleagues (2009) found that, in women from the Framingham Offspring cohort, hip and spine BMD were 5.0 to 8.3 percent greater in postmenopausal women who consumed more than two drinks per day than in nondrinkers. A study of 2,043 postmenopausal women in the United States found that BMD was 3.8 percent higher in women who had more than 29 drinking occasions per month than those who abstained, although this finding only was marginally significant (because of small numbers of daily drinkers) (Wosje and Kalkwarf 2007). Finally, a study in Scotland of 3,218 women aged 50 to 62 found significant increases in BMD in the femoral neck and lumbar spine in women who averaged more than one drink a day, compared with lifetime abstainers (McLernon et al. 2012). However, in general these studies were unable to evaluate effects of heavy drinking, and the processes by which alcohol affects BMD remain uncertain but may involve effects of increased levels of estrogen and calcitonin (Maurel et al. 2012).

In contrast, the prevailing wisdom is that heavy drinking (averaging multiple drinks per day) increases women’s risks of fractures, such as from falls (Epstein et al. 2007). In a combined study of 11,032 women in Canada, Australia, and the Netherlands, the risks of hip fractures and osteoporotic fractures were higher in women averaging two or more drinks a day than in women averaging up to one drink a day (Kanis et al. 2005). In Sweden, a study of 10,902 middle-aged women showed that low-energy fractures were more likely in women who had higher levels of GGT, which is associated with chronic heavy drinking (Holmberg et al. 2006).

## Women’s Drinking and Psychiatric Disorders

### Alcohol Use Disorders

In addition to physical health risks associated with alcohol use, women’s risks of mental health problems also are related to their drinking. It is clear that women’s heavy and binge drinking is associated with alcohol use disorders (AUDs). For example, U.S. data show that among women aged 50 or older, those who engage in binge drinking (four or more drinks on a drinking occasion) have more than three times greater risks of alcohol abuse, and more than five times greater risks of alcohol dependence, than women who drink but do not engage in binge drinking (Chou et al. 2011).

However, there has otherwise been limited attention to gender-specific ways in which women’s drinking may be related to AUDs. One exception is that women, like men, are at greater risk of AUDs if they begin drinking at early ages. A large study in Missouri has found elevated risks of AUDs in women who began drinking before age 18 (Jenkins et al. 2011), confirming findings from U.S. national surveys (Dawson et al. 2008). A second exception is that it has long been thought that development of AUDs is “telescoped” in women compared with men, occurring in a shorter period of time after women begin to drink (Greenfield 2002). However, this pattern was identified in women in treatment for AUDs, and U.S. survey data now indicate that telescoping does not occur in women drinkers in the general population (Keyes et al. 2010) but may be related to the experiences that bring women to treatment.

### Psychiatric Disorders Other Than AUDs

General-population studies often have found links between women’s drinking and psychiatric disorders, but the time order and causes of these linkages are often unclear. For example, a German survey found that women with alcohol abuse or dependence, or women who drank an average of at least 20 to 30 grams of alcohol per day, were more likely than other women to have a variety of psychiatric disorders (affective, anxiety, or somatoform), and the connections between drinking and disorders were stronger for women than for men (Bott et al. 2005). A Danish survey found that any psychiatric disorders were more likely in women averaging more than three drinks a day, and anxiety disorders were specifically more likely among women averaging more than two drinks a day, compared with nondrinkers (Flensborg-Madsen et al. 2011). In addition, U.S. data on women aged 50 and older showed higher risks of both panic disorder and posttraumatic stress disorder (PTSD) in women who engaged in any binge drinking, compared with non–binge drinkers (Chou et al. 2011). Unlike the preceding studies, which linked drinking patterns to increased risks of general psychiatric comorbidity, most studies of women’s alcohol use and psychiatric disorders have focused on comorbidity of specific disorders with AUDs and risky drinking patterns. These more specific linkages are discussed in the sections that follow.

#### Depression

Research clearly has established that depressive disorders and symptoms are more likely among people with AUDs (e.g., Grant et al. 2004), but studies have not always examined this connection specifically among women. However, a large U.S. twin study found that diagnoses of major depression and alcohol dependence were correlated among women (Prescott et al. 2000), and data from the large National Epidemiologic Study on Alcohol and Related Conditions (NESARC) showed that women with major depressive disorder were more likely to report multiple criteria for alcohol abuse and dependence (Lynskey and Agrawal 2008). Research also has repeatedly found associations of women’s depression with binge drinking. For example, in a major Canadian survey, women’s binge drinking (five or more, or eight or more, drinks per day) was associated with measures of recent and longer-term depression (Graham et al. 2007), and data from the large U.S. Behavioral Risk Factor Surveillance System surveys showed that lifetime depression was significantly more likely in women who engaged in binge drinking (four or more drinks in a day) (Strine et al. 2008).

#### PTSD

AUDs often have been associated with symptoms or diagnoses of PTSD. For example, in young adults followed up from the U.S. National Survey of Adolescents, women with PTSD in the past 6 months were more than twice as likely as other women to meet criteria for a *Diagnostic and Statistical Manual of Mental Disorders, 4th Edition* diagnosis of alcohol abuse (Danielson et al. 2009). Among women from the large Missouri Adolescent Female Twin Study, PTSD was associated with a greater likelihood of AUDs (Sartor et al. 2010). In surveys of three Mexican cities, lifetime PTSD was more prevalent in women who misused alcohol (with at least one indicator of alcohol abuse or dependence) (Slone et al. 2006). In addition, in the large California Women’s Health Survey, having symptoms of PTSD doubled the odds that women engaged in binge drinking (Timko et al. 2008). However, most of these studies have not found any effects of PTSD beyond the effects of the traumatic experiences that led to PTSD, a pattern also reported in other recent studies of women who have experienced sexual assaults (Najdowski and Ullman 2009; Testa et al. 2007). Therefore, PTSD may be an indicator of experiences distressful enough to lead women to drink to excess, but PTSD itself may not necessarily be a cause of such drinking.

#### Alcohol and Eating Disorders

Research often has found that eating disorders in women are associated with problem drinking. The strongest recent evidence is in a meta-analysis of 41 studies, mainly in the U.S. and Canada, in which women’s eating disorders consistently were associated with AUDs (Gadalla and Piran 2007). The meta-analysis included a very large Canadian general-population survey in which risks of eating disorders also were associated with heavier weekly drinking among women ages 15 to 44 (Piran and Gadalla 2007). Hypotheses to explain observed links between women’s eating disorders and drinking typically have focused on possible common antecedents (distress, personality characteristics, and genetic factors) rather than on ways that eating disorders might cause or be caused by drinking (Conason and Sher 2006).

The meta-analysis by Gadalla and Piran (2007) showed that problem drinking was associated more specifically with bulimic behavior than with anorexia nervosa. The associations also were stronger among women in community or student samples but were weaker or absent when women in treatment for eating disorders were compared with women in the general population. A multisite European study comparing individuals (mostly women) in treatment versus healthy individuals in the general population also failed to find that those in eating disorders treatment drank more heavily (Krug et al. 2008). It is possible that such negative findings could result because many women receiving treatment or seeking treatment for eating disorders curtail their drinking.

#### Alcohol and Suicidal Behavior

Although research often has reported on factors affecting rates of suicide among women, only rarely have studies been able to show how individual women’s drinking patterns are related to suicidal behavior. An exception was a 20-year follow-up of a large sample of Swedish women hospitalized because of suicidal behavior; those women diagnosed also with alcohol abuse or dependence had a higher risk of later committing suicide (Tidemalm et al. 2008). Most general-population surveys of individual women have shown that suicidal ideation (thinking about committing suicide) was associated with heavier, more frequent, or more hazardous drinking. In the United States, for example, women’s suicidal ideation was associated with hazardous drinking patterns in a longitudinal study of women aged 26 to 54 (Wilsnack et al. 2004) and was associated with alcohol dependence in the large National Longitudinal Alcohol Epidemiologic Survey (Grant and Hasin 1999). A large study of active-duty U.S. Air Force personnel also found that women’s suicidal ideation was associated with higher levels of alcohol problems, but only among women who were not mothers (Langhinrichsen-Rohling et al. 2011). In Seoul, Korea, women aged 18 to 64 showed a strong association of suicidal ideation with drinking nearly daily (Park et al. 2010). Finally, a French survey of women aged 18 to 30 found that suicidal ideation was more common in heavier drinkers, although the relationship no longer was statistically significant after controlling for effects of depression and other adverse experiences (Legleye et al. 2010).

## Alcohol-Related Injuries

Similar to research on women’s suicidality, research on women’s alcohol-related injuries has given more attention to gender differences in injury rates and how women’s injury rates are related to population drinking patterns and less attention to how drinking is related to the risks of injury in individual women. However, studies have reported two consistent findings about how individual drinking patterns are linked to injuries.

First, risks of injury increase among women who have consumed alcohol in the 6 hours before being injured; women’s injury risks associated with drinking occur relatively rapidly. This conclusion has been confirmed by a combined analysis of 28 hospital emergency-department studies in 16 countries (Borges et al. 2006). Additional confirmation has come from a large emergency-department survey in Sydney, Australia, where the risk was greatest in women who had consumed more than 90 grams of alcohol in the 6 hours before being injured (Williams et al. 2011).

The other consistent finding is that risks of injury are greatest among women whose drinking patterns are particularly heavy or hazardous. A study of women outpatients at a Veterans Administration hospital found that the likelihood of multiple recent injuries was nearly doubled in the heaviest versus the lightest drinkers (Chavez et al. 2012). A study of women with high-risk drinking patterns at five U.S. colleges found that their risks of recent injury were directly related to their number of days of drinking five or more drinks (Mundt et al. 2009). In addition, large surveys of women aged 45 to 69 in three Eastern European countries found that the percentage of women with injuries was higher in women with high scores on the CAGE^3^ screening instrument for problem drinking (Vikhireva et al. 2010).

## Intimate Partner Violence

Associations between alcohol use and intimate partner violence (IPV) have been well documented in research in North America. Male-to-female IPV perpetration consistently has been linked to heavy and problem drinking by men (Caetano et al. 2000; Thompson and Kingree 2006). The large-scale NESARC survey found that past-year IPV victimization was more likely in women who have symptoms of alcohol abuse or dependence (La Flair et al. 2012), and meta-analysis of six surveys of adolescents and young adults showed that women’s frequency and/or quantity of drinking was positively related to their perpetration of IPV (Rothman et al. 2012). Furthermore, a comparative study of alcohol consumption and IPV in Canada, the United States, and eight countries in Latin America found that in all 10 countries, rates of physical partner aggression were higher among drinkers than nondrinkers (men and women); and among drinkers, rates were higher among persons who reported drinking larger amounts per occasion. Women reported being victims of more severe aggression than men, and men were more likely than women to be drinking at the time of an incident of physical aggression (Graham et al. 2008). Other multinational studies have shown that odds of IPV were greater where one or both partners had alcohol problems (Abramsky et al. 2011) and that aggression severity was significantly higher if one or both partners had been drinking when the aggression occurred (Graham et al. 2011). However, in all this research, it is unclear to what extent drinking is a cause or an effect of IPV, or both.

## Alcohol and Sexual Assault

It has been known for some time that women’s drinking is positively associated with their risks of sexual assault, but how and why this association occurs remains unsettled (Abbey et al. 2004). Part of the association results because women often drink with men who drink, and the men’s intoxication makes them more likely to be sexually aggressive toward women (Abbey 2011). Other links between women’s drinking and sexual assaults are harder to interpret because investigators often lack time-ordered data, they differ in the types of sexual activity they evaluate (ranging from rape to much broader categories of unwanted sexual advances), and most of their studies are limited to college women (as a high-risk group).

Nevertheless, certain patterns have become clear in recent years. First, risks of sexual assault are most clearly higher in women who have established patterns of binge drinking or problem drinking. For example, in a large national survey of college women in 1999, women with alcohol problems were more likely to report experiencing unwanted sexual advances (Pino and Johnson-Johns 2009). At a large New York State university, women who increased their drinking during their first year in college (and who averaged more than four drinks per drinking occasion, with frequent such occasions) had higher odds of sexual victimization (Parks et al. 2008).

Second, women are more likely to experience rape or other severe sexual assault if they become intoxicated, at the time of the assault or as a typical drinking pattern. A large U.S. survey of college women found that the percentage who had been raped was high in women with any recent experience of binge drinking (four or more drinks per occasion) and that more than two-thirds of the women who had been raped reported being intoxicated at the time (Mohler-Kuo et al. 2004). A study of more than 300 young women who had been sexually assaulted since age 14 found that the odds of sexual penetration were greater only among women reporting high levels of intoxication (Testa et al. 2004). An earlier national survey of college women who had experienced sexual victimization found that the severity of the assault was predicted in part by the women’s frequency of intoxication (Ullman et al. 1999).

Findings like these have led some investigators to conclude that one reason why drinking may increase women’s risks of sexual assault is that highly intoxicated women may be incapacitated, unable to resist unwanted sexual advances. A national survey of college women found that a past-year history of binge drinking (five or more drinks at a sitting) was specifically associated with experiencing incapacitated rape (McCauley et al. 2009). A study of first-year college students found that reported maximum consumption per occasion during the fall semester was strongly associated with experiencing incapacitated rape (Testa and Hoffman 2012). A number of related studies reviewed by Testa and Livingston (2009) led to the conclusions that in many rapes, especially of college students, women are incapacitated by some form of substance use, and that many rapes associated with alcohol use involve incapacitation.

## Conclusions

Because alcohol consumption has become a more normal activity for women, it is important for women to have science-based information to help them decide whether and when to drink, and in what amounts, based on potential risks or benefits of drinking. Such past and current information has had some important limitations. Some of these limitations have been addressed in recent decades. In most recent studies (e.g., Mukamal et al. 2010; Patra et al. 2010), apparent health benefits of moderate drinking now are based on comparisons with lifetime abstainers, excluding potentially sicker ex-drinkers who were part of some earlier comparisons. Also, long-term studies of alcohol consumption in women now are likely to include more detailed measures of baseline drinking (Moore et al. 2005; Wilsnack et al. 2006) than earlier studies used (Stampfer et al. 1988). However, some research findings are still presented in terms of rates of health outcomes in whole *groups* of women (such as for injuries and suicidality; Landberg 2010; Ramstedt 2005), which can be misleading if these results are used to draw conclusions about the effects of drinking on *individuals*. Finally, research on long-term health effects of women’s drinking can measure only some of the lifestyle characteristics (such as eating patterns and exercise) that may be associated with how women drink and that may account for some of the apparent effects of drinking (Mukamal et al. 2010; Rimm & Moats 2007).

A major current limitation of information about alcohol effects is that such effects often are reported, in scientific papers but particularly in the news media, as simple associations (this drinking pattern is associated with that health outcome). Less is said about how large the effects are (not very large for some cardiovascular benefits of moderate drinking), and adverse effects often are implied to increase in a linear way with each unit increase in drinking. There is too little attention paid to how the effects of drinking may not be linear (with the exception of research on cardiovascular benefits versus hazards at different levels of drinking). There also is too little attention paid to how drinking may be both a cause and an effect of some adverse health and behavioral outcomes (such as psychiatric disorders and intimate partner violence). Finally, research findings often are presented as if they applied similarly to all women drinkers, without discussing how other conditions and contexts (such as a drinker’s other health conditions) might modify how alcohol affects health. (One exception, for example, is the research by Liu et al. [2010] showing that risks of cirrhosis from relatively heavy drinking are greater in women with high body mass indices.) Therefore, what we should strive for is information about health effects of women’s drinking that shows not only the effect sizes, but also when and where and among which women the effects are greatest.

Keeping those limitations in mind, the findings summarized here may offer some guidelines for women making personal decisions about drinking in midlife. Light-to-moderate drinking is associated to some extent with reduced risks of some cardiovascular problems, strokes, and weakening of bones. On the other hand, even low levels of alcohol consumption may cause women some increase in risks of breast cancer and liver problems, and heavy drinking also increases risks of hypertension and bone fractures and injuries. Women’s heavy drinking patterns and AUDs are associated with increased likelihood of many psychiatric problems, including depression, PTSD, eating disorders, and suicidality. Women’s heavy drinking and AUDs also are associated with increased risks of intimate partner violence and sexual assault, although causality in the associations of drinking with psychiatric disorders and with violence remains unclear. On balance, the evidence summarized here suggests that, for those women who choose to drink, moderation in consumption is the safest or least costly strategy to adopt toward alcohol.

## Figures and Tables

**Figure:**
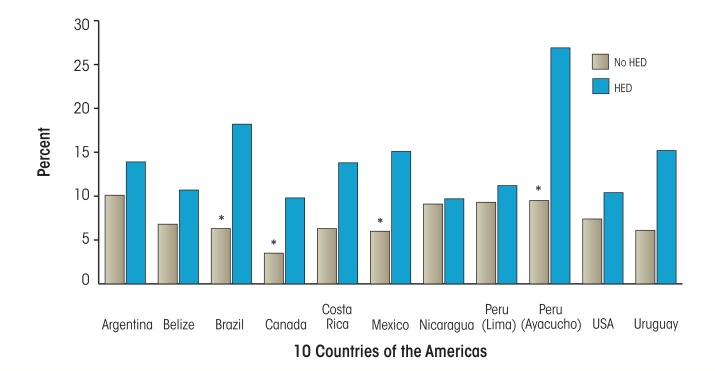
Female intimate-partner violence victimization by women’s past-12-month heavy episodic drinking (HED) (10 countries of the Americas). NOTE: * p < .05 for logistic regression, controlling for age. SOURCE: Graham, K.; Bernards, S.; Munné, M.; and Wilsnack, S.C.; Eds. *Unhappy Hours: Alcohol and Partner Aggression in the Americas.* Washington, DC: Pan American Health Organization, 2008.

## Footnotes

1 Because women’s bodies generally have less water than men’s bodies, alcohol becomes less diluted, and women therefore reach higher blood alcohol levels than men even if both are drinking the same amount.

2 Former drinkers reported drinking in the past but not in the last 12 months.

3 The CAGE is a screening instrument (Ewing 1984) consisting of the following four questions: Have you ever felt you should cut down on your drinking? Have people annoyed you by criticizing your drinking? Have you ever felt bad or guilty about your drinking? Eye opener: Have you ever had a drink first thing in the morning to steady your nerves or to get rid of a hangover? Two positive responses are considered a positive test and indicate further assessment is warranted.
